# Analysis of the Impact of Agriculture and Logging on Forest Habitat Structure in the Ankasa and Bia Conservation Area of Ghana

**DOI:** 10.1002/ece3.70712

**Published:** 2024-12-12

**Authors:** George Ashiagbor, Sinka Khadijah Abubakar, Sandra Sawdiatu Inusah, Abena Owusu Adjapong, Gideon Nyamekye Osei, Prosper Basommi Laari

**Affiliations:** ^1^ Department of Wildlife and Range Management Kwame Nkrumah University of Science and Technology (KNUST), PMB Kumasi Ghana; ^2^ Department of Ecotourism, Recreation and Hospitality University of Energy and Natural Resources, PMB Sunyani Ghana; ^3^ Department of Environment and Resource Studies Simon Diedong Dombo University of Business and Integrated Development Studies Wa Ghana

**Keywords:** fragmentation analysis, landscape ecology, protected areas in Ghana, remote sensing

## Abstract

Ghana's Ankasa (ACA) and Bia Conservation Area (BCA) are experiencing forest loss due to agricultural conversions. However, there is limited comprehensive analysis of these conversions and their impact on the forest habitat structure in these areas. This study aims to analyse anthropogenic‐induced forest habitat loss and fragmentation in the ACA and BCA. Landsat images for the epochs 1980, 2000 and 2020 were pre‐processed, and subsets were created using a 5 km buffer of the two conservation areas. The images were classified into forest, agriculture and built‐up. The classified images were analysed for landscape pattern changes using patch density (PD), edge density (ED), largest patch index (LPI), landscape shape index (LSI) and aggregation index (AI). The Wilcoxon signed‐rank test was used to analyse changes in landscape structure. The results indicate that forest cover in the ACA decreased by 16.4% from 100,941.6 ha in 1980 to 84,410.6 ha in 2020, and in the BCA, it decreased by 14.4% from 70,211.8 to 60,117.36 ha. There was no encroachment from agricultural activities within the conservation areas, but agricultural activities, mainly cocoa expansion, increased within the 5 km buffer, leading to the decline in forest cover. The landscape analysis shows that the forest patches have become fragmented, disjointed and isolated, especially within the 5 km buffer. This is indicated by increased PD, decreased AI, decreased LPI and increased ED. The immediate loss of forest habitat cover in the off‐reserve landscape and the significant levels of forest fragmentation, resulting in the loss of forest connectivity, have significant implications for wildlife conservation. Ecological restoration and conservation efforts are needed to reduce this potential impact. Ecologists have recommended transitioning from monoculture cocoa to cocoa agroforestry to improve forest habitat connectivity within adjoining cocoa farms in the landscapes of these conservation areas.

## Graphical abstract

Ghana's Ankasa and Bia Conservation Areas are facing rapid forest loss due to the expansion of agricultural activities, leading to widespread land conversion. As a result, forest patches on the outskirts of these protected areas have become fragmented, isolated and disconnected. This immediate threat has consequences for biodiversity and wildlife conservation, requiring prompt action and attention.

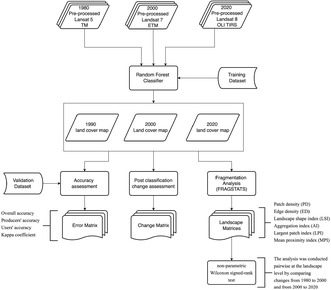

## 1 Introduction

The Ankasa Conservation Area (ACA) and the Bia Conservation Area (BCA) in Ghana, along with their surrounding areas are experiencing forest loss through land conversion resulting from increasing agricultural activities (Chen et al. 2021; Dewan, Yamaguchi, and Rahman 2012). These human‐induced land cover changes have impacted the ecology of these conservation areas, including ecological interactions and processes. This has led to significant landscape degradation, habitat destruction and species extinction (Dewan, Yamaguchi, and Rahman 2012). Also, conflicts between humans and wildlife have increased drastically in the ACA and BCA and are attributed to these land‐use changes (in this case, cocoa expansions and illegal logging) and increasing human populations (Sam et al. 2005; Harich et al. 2013). Therefore, it is vital to understand how human activities impact forest cover in and around protected areas so that these regions and their ecological systems can be effectively managed using scientific methods (With 2019).

However, studies on the ACA and BCA landscapes have mainly focused on the community, population and behavioural ecology of species (Bempah, Dakwa, and Monney 2019; Bumpi and Kitone 2009; Danquah et al. 2001; Danquah and Oppong 2013; Seidu et al. 2019). Other studies include population trends of forest elephants ( *Loxodonta cyclotis* ) (Danquah et al. 2001; Danquah and Oppong 2013; Sam et al. 2005), the presence of white‐naped mangabey *Cercocebus lunulatus* (Nolan et al. 2019), the richness and diversity of bats (Holbech 2013), ornithological studies (Dyer 1997) and land use land cover changes (Asare et al. 2014; Ashiagbor et al. 2020). Some studies have also focused on the environmental requirements of elephants in the BCA Sam et al. (2005), human–wildlife conflicts (Sam et al. 2005; Harich et al. 2013) and biological surveys estimating the abundance of wildlife and plants in the Bia Biosphere Reserve (MAB National Secretariat 2014).

A comprehensive analysis of the landscape structure (composition and configuration of landscape elements) in and around protected areas in Ghana and its relationship with anthropogenic conversions, particularly agricultural expansion, is limited. Also, there is a lack of spatio‐temporal understanding of landscape changes and anthropogenic impact dynamics. Biodiversity is closely tied to habitats, which consist of various landscape elements arranged spatially at different scales. Changes in the composition and spatial arrangement of these elements can have varying effects on ecological systems (Walz 2011). Therefore, understanding changes in landscape structure is crucial for protection and planning, as ecological processes are heavily influenced by it (Walz 2011). Furthermore, landscape structural studies can offer valuable insights for policymaking and resource management. Quantifying the variations in habitat and landscape structure is essential to assist in wildlife management and conservation efforts in landscapes.

This paper aims to analyse how human activities, mainly agricultural expansion, have altered the landscape structure in the ACA and BCA and discusses the implications of this transformation on conservation. It answers the question, how have human activities, mainly agricultural expansion, transformed forest habitats of the BCA and ACA and their catchment? The specific objectives are (1) to analyse changes in forest habitat cover within the ACA and BCA, including a 5 km radius of the conservation area from 1980 to 2020 and (2) to analyse changes in the landscape structure of the ACA and BCA, at the class and landscape levels from 1980 to 2020.

## 2 Method

### 2.1 Study Area

**FIGURE 1:**
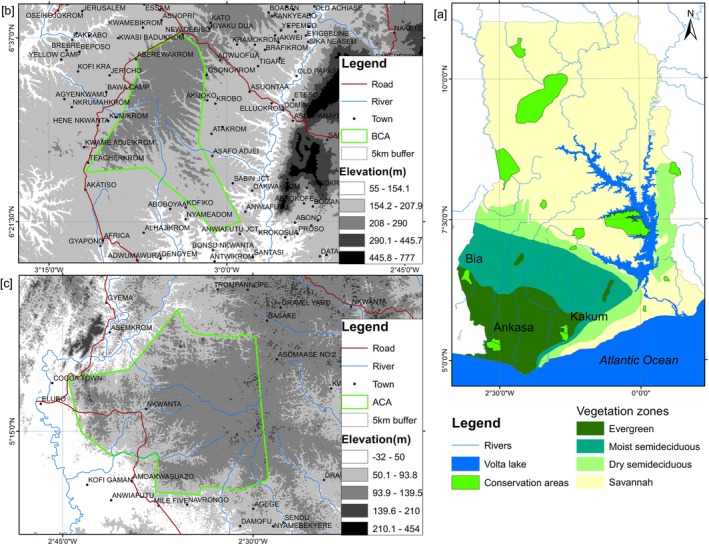
(a) Vegetation zone map of Ghana showing the distribution of conservation areas, (b) Bia conservation area and (c) Ankasa conservation area. Elevation overlay is SRTM 1 arc‐second global dataset downloaded from https://earthexplorer.usgs.gov/.

There are three conservation areas in the forest agroecological zone of Ghana, that is, the Ankasa, Bia and Kakum conservation areas. This study was conducted in the ACA and BCA (Figure 1). The ACA and BCA were chosen because of the availability of cloud‐free Landsat dataset for the epochs under investigation. Bailey et al. (2016) emphasised that conservation areas should not be analysed in isolation, as they are influenced by the surrounding landscape's land use, species and ecological processes. Therefore, a buffer of 5 km was created around the conservation area's boundary to define the study area. The reason for choosing this distance is that the Ghana Wildlife Division considers communities and related activities within a 5 km radius of conservation areas for management purposes. Additionally, the 5 km buffer zone around conservation areas is adequate for assessing the direct impact of anthropogenic drivers on conservation areas. The study area was divided into two regions: the regions inside the conservation area's boundaries, hereafter referred to as ‘on‐reserve’ areas and delineated by the boundaries of the conservation areas. The ‘off‐reserve’ represents the areas outside the delineated boundaries of the conservation areas but within the 5 km buffer.

#### 2.1.1 The Ankasa Conservation Area (ACA)

The ACA is located at latitude 5°17′ N and longitude 2°39′ W in the Jomoro District, southwest of Ghana, on the border of Ivory Coast (Ghana Wildlife Division 2000; IUCN/PACO 2010). It is an ancient tropical evergreen rainforest with the highest botanical diversity in Ghana (Davis and Philips 2005). The rainfall is bimodal, occurring from April to July and September to November with an average of 1700–2000 mm yearly rainfall, making it Ghana's wettest region (Belelli Marchesini et al. 2008; Rödel et al. 2005). The ACA has a diverse collection of over 800 vascular plant species, such as Dahoma (*Piptadeniastrum africanum*), Makore (*Tieghemella africana*) and Khaya ( *Khaya senegalensis* ) (Belelli Marchesini et al. 2008; Owusu et al. 2022; Ghana Wildlife Division 2000). It is also home to many well‐known plants, such as Marantas (*Maranta leuconeura*), Bloody Lilly ( *Scadoxus multiflorus* ) and Glory bower (*Clerodendrum*) (Belelli Marchesini et al. 2008). In addition, the ACA houses 10 different primate species, some of which are endangered, such as the Diana monkey ( *Cercopithecus diana* ) and West African chimpanzee, as well as large and charismatic mammals like the bongo (*Tragelaphus euryceros*), yellow‐backed duiker (*Cephalophus silviculture*), leopard ( *Panthera pardus* ) and forest elephant ( *Loxodonta cyclotis* ) (Ghana Wildlife Division 2000; Owusu et al. 2022). Furthermore, it is home to 600 butterfly species and has a diversified avifauna (IUCN/PACO 2010).

#### 2.1.2 The Bia Conservation Area (BCA)

The BCA is a 306 km^2^ area in south‐western Ghana between longitudes 6°20′–6°40′ N and latitudes 3°00′–3°10′ W. The yearly rainfall varies from 1500 to 1800 mm. There are two main periods of rain: the major in May/June and the minor in September/October. The dry season lasts from December to March. The temperature ranges from 24°C to 28°C monthly. The terrain is generally undulating, with elevations between 200 and 550 m (Danquah 2016). The BCA has the third‐highest forest elephant population in Western Africa (Harich et al. 2013), making it an area of conservation significance for forest elephants. The BCA protects three species of primates, including chimpanzees ( *Pan troglodytes verus* ), olive colobuses ( *Procolobus verus* ) and Geoffroy's pied colobuses ( *Colobus vellerosus* ). Additionally, the BCA also protects carnivores such as the Tree Pangolin ( *Dendrohyrax dorsalis* ), Leopard ( *Panthera pardus* ), Golden Cat (*Profelisaurata*) and two ungulates, the Forest Elephant (*Loxodonta africana cyclotis*) and Bongo (*Tragelaphus euryceros*). Some species of the family Accipitridae (birds of prey), including the Hooded Vulture ( *Necrosyrtes monachus* ), Black Sparrowhawk ( *Accipiter melanoleucus* ), African Goshawk ( *Accipiter tachiro* ), Black Kite (*Milvusmigrans*) and African Hobby ( *Falco cuvierii* ) with high protection value in the country can be found in the area (MAB National Secretariat 2014).

### 2.2 Satellite Data Acquisition, Processing and Classification

**FIGURE 2:**
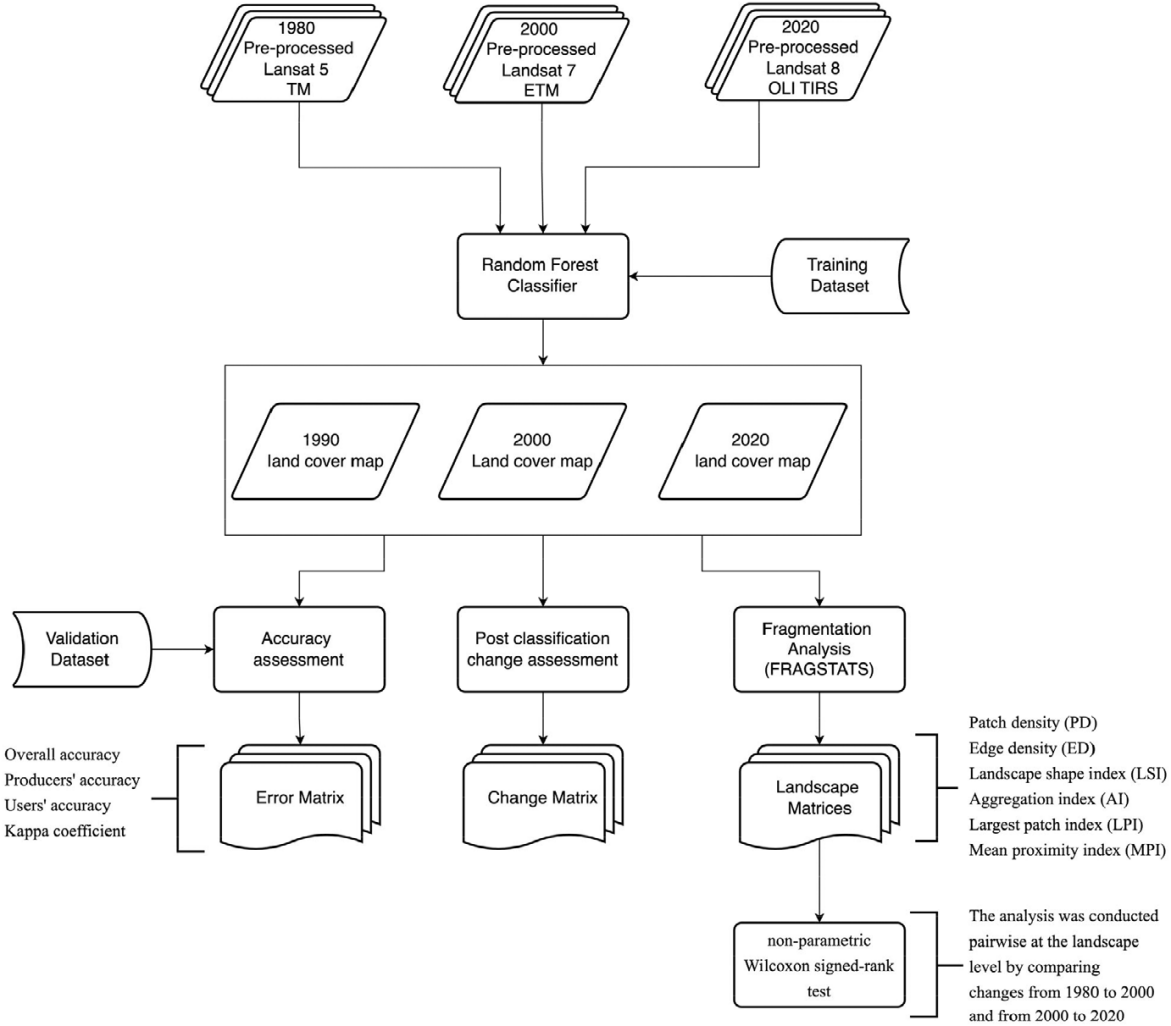
The methodological flowchart for data analysis used in this study.

The methodological flowchart for data analysis is illustrated in Figure 2.

The Landsat collection 2 level 2 satellite images of the study area (image scenes; P195/R55 and P195/R56) for the epochs 1980, 2000 and 2020 were obtained from the United States Geological Survey website (https://earthexplorer.usgs.gov/). See Table 1 for the detailed list of Landsat satellite image scenes used in this study. The Landsat images covered three time steps spanning up to 20 years. Landsat data availability and quality considerations, such as the availability of cloud‐free scenes, informed the selection of the images. Similarly, the 2000 image was selected based on quality and time lag to observe intermediate trends between the 40‐year change period. The image bands were processed to surface reflectance to correct the differences in sensor dates. The image bands for each year were stacked and mosaicked, and the study areas were extracted using the study area shapefiles. A reconnaissance survey and a review of related works were conducted in the study area to identify potential land use and land cover (LULC) classes. Within the context of this research, five land cover classes were identified. These include:

1. Closed canopy forest class represents land areas covered by primary and secondary woody vegetation with a crown canopy of over 60% and a minimum height of 5 m. These areas are mainly found inside the boundaries of conservation areas and include intact forest areas. This class also includes a large group of tropical African palm trees ( *Raphia farinifera* ) in lowland riparian and swamp zones around the ACA.
2. Open canopy forest class includes degraded forests resulting primarily from logging activities. The crown cover in this class ranges from 15% to 60%, representing human activities in the landscape through logging.
3. Tree crops class represents land areas mainly dominated by cocoa farms ( *Theobroma cacao* ) and some patches of rubber plantations ( *Hevea brasiliensis* ) around the ACA. The cocoa class represents a major anthropogenic land use conversion as cocoa is considered the major driver of deforestation in the forest ecological zone of Ghana.
4. Food crops, which also represent anthropogenic impact in the study landscape, mainly through subsistence crops farming such as cassava ( *Manihot esculenta* ), yam ( *Dioscorea cayenensis* ), cocoyam ( *Colocasia esculenta* ), maize ( *Zea mays* ), plantain (*Musa paradisiaca*) and banana ( *Musa acuminata* ).
5. Other land cover classes, mainly represent human settlement areas.

A survey was conducted using a Global Positioning System (GPS) receiver to collect coordinates for the selected land use and land cover classes. Sampling points for the classes were extracted from high‐resolution Google Earth images to complement the field sampling efforts. At least 50 training points were collected for each class and distributed evenly across the study areas. Seventy percentage of the coordinate points collected were used as training data, with the remaining 30% used for validation. The pixel‐based non‐parametric machine‐learning Random Forest classification algorithm was used to classify the images into the land cover categories using the ‘random forest’ package in R software. The random forest classifier was chosen because it has been shown to yield a higher classification algorithm in the land cover mapping of Ghana's forest landscape compared to other classifiers (Ashiagbor et al. 2023). The error matrix was then calculated by overlapping the validation points on the resulting classified image. The error matrix was then used to compute the final LULC maps' overall accuracy, producer accuracy, user accuracy and Kappa coefficient.

### 2.3 Characterisation of Landscape Structure Using Metrics

Six complementary landscape metrics were used to characterise the structure (i.e., composition and configuration) of each landscape. The six metrics include patch density (PD), edge density (ED), landscape shape index (LSI), aggregation index (AI), largest patch index (LPI) and mean proximity index (MPI). PD is the number of patches per 100 ha (1 km^2^) and ranges from 0 (Forman 1995; Turner and Gardner 2015; Mathur and Mathur 2010; Asabere et al. 2020). A higher PD indicates fragmentation, or the division of a class into multiple patches. The ED, measured in metres per hectare with a minimum value of 0, which rises with increasing fragmentation and shape complexity, provides valuable information about the composition and configuration of a landscape (Asabere et al. 2020). The LSI compares the complexity of spatial object shapes to standard geometric objects such as a square or circles. It increases as the shape becomes more complex than a standard geometric unit. Increased LSI indicates increased fragmentation (Asabere et al. 2020; Forman 1995; Turner and Gardner 2015). The AI, which ranges from 0 to 100, measures how much the landscape is aggregated. AI is zero when the patches are wholly separated (no similar adjacent patches) and increases as the landscape becomes more grouped, reaching 100 when it becomes a single patch (Meng, Cai et al. 2023). The LPI represents the proportion of the entire landscape the largest patch occupies. The MPI measures isolation and fragmentation. This value increases as the area within the specified search radius becomes more occupied by patches of the same type, as those patches become closer and more contiguous and as their distribution becomes less fragmented. A low MPI indicates isolation, whereas a high MPI indicates proximity (Forman 1995; Turner and Gardner 2015). The landscape metrics were calculated using the spatial statistical tool FRAGSTATS (version 3.3). The categorical LULC raster for 1980, 2000 and 2020 of the BCA and ACA were used as input data sources for the landscape structure analysis.

### 2.4 Analysis of Changes in Landscape Structure

The post‐classification change matrix was utilised to examine human‐induced changes in forest cover. The non‐parametric Wilcoxon signed‐rank test was used to analyse statistically significant differences (*p* < 0.05) in landscape structure. The analysis was conducted pairwise by comparing changes from 1980 to 2000 and 2000 to 2020. The analysis was conducted at the landscape level. This was done by randomly subsetting 30 1 km × 1 km paired sample grids from the 1980, 2000 and 2020 landcover maps. The six landscape metrics, PD, ED, LSI, AI, LPI and MPI, were calculated for each grid, and the changes in metrics were compared statistically.

## 3 Results

### 3.1 Accuracy of the Land Use and Land Cover Maps

The 1980 land cover map of the ACA achieved an overall accuracy of 78.7% and a kappa value of 0.73, indicating good agreement, as shown in Table 2. Similarly, the 2000 and 2020 LULC maps had overall accuracies of 82.2% and 90.3%, respectively, and kappa values of 0.77 and 0.88, respectively. On the other hand, the 1980 LULC map of the BCA had an overall accuracy of 77.5% and a kappa value of 0.71, also indicated in Table 1. The 2000 and 2020 LULC maps of the BCA achieved overall accuracies of 81.4% and 90.7%, respectively. These accuracies were deemed sufficient for the study.

**TABLE 1:** List of Landsat image scenes used in the study.

Epoch	Path/row	Sensor ID	Image ID	Date acquired
1980	195/055 195/056	TM	LT05_L2SP_195055_19860118_20200918_02_T1 LT05_L2SP_195056_19860118_20200918_02_T1	1986/01/18
2000	195/055 195/056	ETM	LE07_L2SP_195055_20000202_20200918_02_T1 LE07_L2SP_195056_20000202_20200918_02_T1	2000/02/02
2020	195/055 195/056	OLI_TIRS	LC08_L2SP_195055_20200405_20200822_02_T1 LC08_L2SP_195056_20200405_20200822_02_T1	2020/04/05

**TABLE 2:** LULC accuracy report for the Ankasa and Bia Conservation area for 1980, 2000 and 2020. (UA = Producers' Accuracy and UA = Users' Accuracy).

	1980		2000		2020	
	PA (%)	UA (%)	PA (%)	UA (%)	PA (%)	UA (%)
Ankasa Conservation area (ACA)						
Closed‐canopy forest	74.14	75.44	77.59	78.95	86.21	92.59
Open‐canopy forest	70.9	72.22	74.55	78.85	85.45	92.16
Tree crop	76.32	69.05	78.95	73.17	89.47	91.89
Food crop	87.01	95.71	88.31	94.44	94.81	84.88
Others	83.33	71.43	93.33	77.78	96.67	96.67
Overall accuracy	78.70		82.20		90.30	
Kappa	0.73		0.77		0.88	
Bia Conservation area (BCA)						
Closed‐canopy forest	72.41	72.41	75.86	78.57	87.93	98.08
Open‐canopy forest	72.73	72.73	72.73	76.92	87.27	90.57
Tree crop	83.78	67.39	81.58	72.09	92.11	87.50
Food crop	83.12	95.52	89.61	94.52	93.51	87.80
Others	74.19	71.88	86.67	76.47	93.33	90.32
Overall accuracy	77.50		81.40		90.70	
Kappa	0.71		0.76		0.88	

### 3.2 Forest Cover Changes in the Ankasa and Bia Conservation Area

#### 3.2.1 Ankasa Conservation Area

**FIGURE 3:**
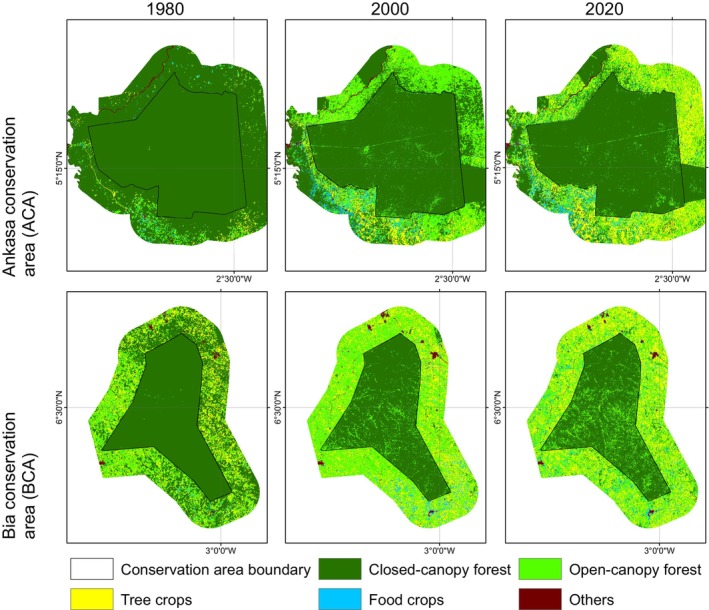
Land use land cover maps for the ACA and BCA for the epoch 1980, 2000 and 2020. NB: The line observed crossing over the ACA conservation area is a high‐tension power line, which is regularly maintained by clearing the trees underneath.

**FIGURE 4:**
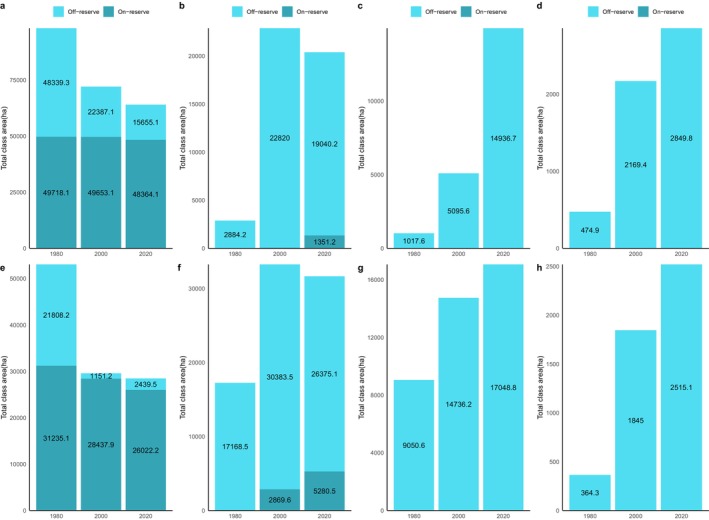
Changes in land use land cover in the ACA and BCA from 1980 to 2000 and from 2000 to 2020. Figures a, b, c and d represent changes in closed‐canopy forests, open‐canopy forests, tree crops and food crops, respectively, in the ACA. Figures e, f, g and h represent changes in closed‐canopy forests, open‐canopy forests, tree crops and food crops, respectively, in the BCA.

In 1980, closed‐canopy forests covered 98,057.4 ha of the ACA. Of this, 49,718.1 ha (50.7%) were within the conservation area boundaries, with the remaining 49.3% in the off‐reserve areas (Figures 3 and 4). Between 1980 and 2000, closed‐canopy forests decreased to 72,040.2 ha, marking a 26.6% loss. Subsequently, closed‐canopy forests further declined to 64,019.2 ha. In contrast, open‐canopy areas expanded from 2884.2 ha to 22,820 ha between 1980 and 2000, but then decreased to 20,391.4 ha by 2020, with 1351.2 ha within the conservation area and 19,040.2 ha in off‐reserve areas.

During the first change period, the increase in open‐canopy forests in the off‐reserve regions is linked to human activities such as logging, which has transformed closed‐canopy forests into open‐canopy ones. Figure 4a illustrates that the loss of closed‐canopy forests mainly occurred in the off‐reserve regions. However, during the second change period, open‐canopy forests decreased due to agricultural conversions in the off‐reserve regions. The study area saw an increase in agricultural conversions to food crops and tree crops, particularly in the off‐reserve regions. No food crops and tree crops were identified within the boundaries of the ACA (on‐reserve area). Tree crops, particularly cocoa and rubber, expanded from 1017.6 to 5095.6 ha between 1980 and 2000. The area under tree crop plantation increased from 5095.6 to 14,936.7 ha between 2000 and 2020. Food crops increased from 474.9 to 2849.8 ha over the 40‐year study period (Figures 3 and 4). The rise in agricultural activities in the off‐reserve landscape led to a decline in closed‐canopy and open‐canopy forests in those areas.

#### 3.2.2 Bia Conservation Area

In the BCA, the closed‐canopy forest decreased from 53,043.3 ha in 1980 to 29,589.1 ha in 2000, with the change primarily occurring in the off‐reserve regions (Figures 3 and 4). The decline in closed‐canopy forests led to an increase in open‐canopy forests in the off‐reserve areas. By 2020, closed‐canopy forests in the off‐reserve regions had decreased to 2439.5 ha from the initial 21,808.2 ha in 1980. Open‐canopy forest cover, however, increased from 17,168.5 to 26,375.1 ha between 1980 and 2020. The increase predominantly took place in the off‐reserve regions. No tree crops or food crops were mapped within the boundaries of the BCA. This suggests that the observed increase in agricultural activities occurred in the off‐reserve regions, leading to a loss of closed‐canopy and open‐canopy in the region. Tree croplands increased from 9050.6 to 17,048.8 ha between 1980 and 2020. Additionally, food crop areas increased from 364.3 ha in 1980 to 2415.1 ha in 2020 (Figures 3 and 4).

### 3.3 Changes in Landscape Patterns in the ACA and BCA and Their Catchment

In the following sections, the results of the landscape structural analysis are presented at two levels: (1) the landscape level and (2) the class levels. The class level changes show the dynamics of anthropogenic conversions (agricultural and logging activities) on forest spatial structure of forest cover in the study landscape. The metric‐based landscape characterisation analyses are presented for each study area.

#### 3.3.1 Ankasa Conservation Area

**FIGURE 5:**
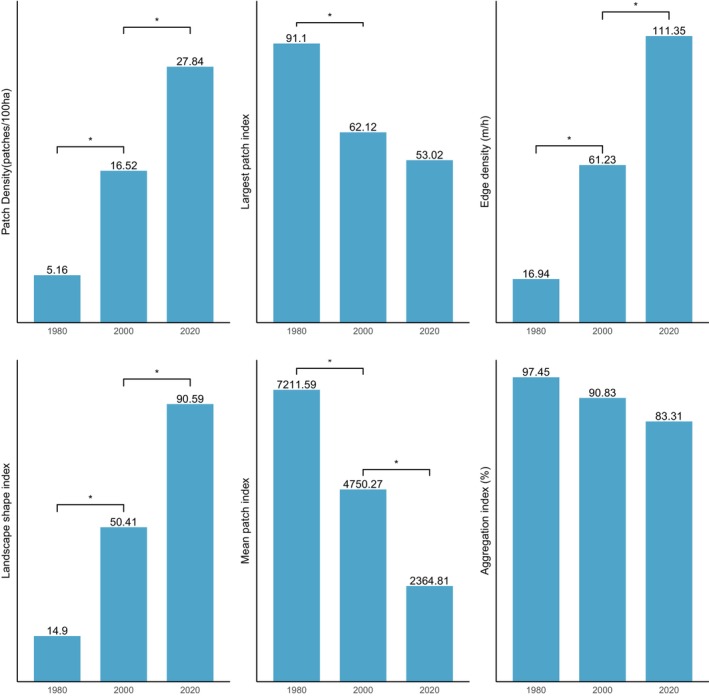
Changes in landscape structural metrics in the Ankasa conservation areas (ACA) from 1980 to 2020. The bars indicate the results of the Wilcoxon signed‐rank test. * show statistically significant differences in landscape metrics at *p* < 0.05.

Figure 5 shows the results of changes in landscape level in the ACA from 1980 to 2000. In the ACA study area, the PD of focal classes increased from 28.02 patches/100 ha to 30.4 patches/100 ha between 1980 and 2000 and increased again from 39.58 patches/100 ha by 2020, which indicates a division of the classes into multiple patches. AI decreased from 84.5% in 1980 to 72.76% in 2020 and LPI also reduced from 58.6 to 32.45 between 2000 and 2020. This shows that focal and dominant classes in the ACA have been subdivided into smaller and fragmented patches with increasing levels of fragmentation across the landscape. The shapes of dominant classes in the ACA have become more irregular and complex, and this has been characterised by the continual increase in ED and LSI. ED increased significantly from 103.36 to 181.62, and LSI increased from 74.55 to 130 (Figure 5). The MPI, however, increased from 3698.47 to 7058.16, indicating that the dominant class in the landscape remains more contiguous and less fragmented. In summary, the results indicate a landscape experiencing fragmentation, with the focal patches becoming isolated, complex and irregular.

**FIGURE 6:**
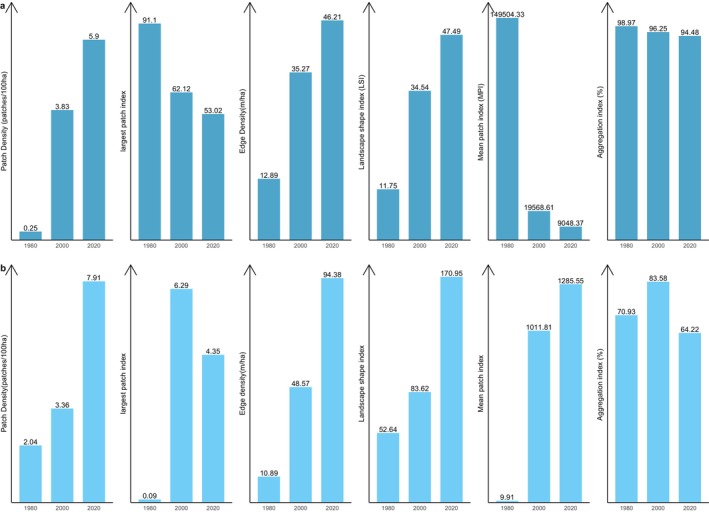
Structural changes in the closed‐canopy and open‐canopy forest classes in the ACA from 1980 to 2020. The set (a) closed‐forest class and (b) open‐forest class.

**FIGURE 7:**
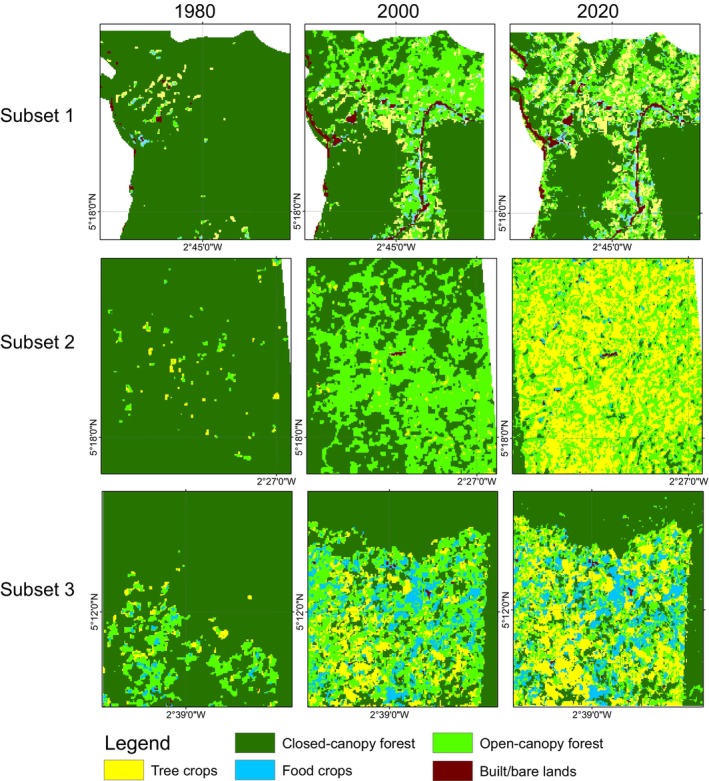
Reduction and fragmentation of forest in the ACA between 1980 and 2020 mainly driven by anthropogenic agricultural expansion.

Figure 5 shows changes in structure in the closed‐canopy and open‐canopy forest classes as an indicator of the impact of agricultural expansion on the structure of forest cover in the ACA. Closed‐canopy forests experienced a significant increase in PD from 0.25 patches/100 ha in 1980 to 5.9 patches/100 ha. This shows that the closed‐canopy forest class in the ACA has been subdivided into smaller and fragmented patches. The LPI for the closed canopy forest class was reduced from 91.1 to 53.02 in the 40‐year study period. ED, LSI and AI increased significantly from 1980 to 2020 (Figure 6). This shows that the closed‐forest class in the ACA has become fragmented, and the patches have become irregular and complex in shape as a result of agricultural expansion. A visual inspection of the land cover classes shows how human agricultural activities have fragmented the forested landscape, mainly cocoa, rubber and food crops (Figure 7). The forest class has significantly reduced, and the patches have become isolated and disjointed.

#### 3.3.2 Bia Conservation Area

**FIGURE 8:**
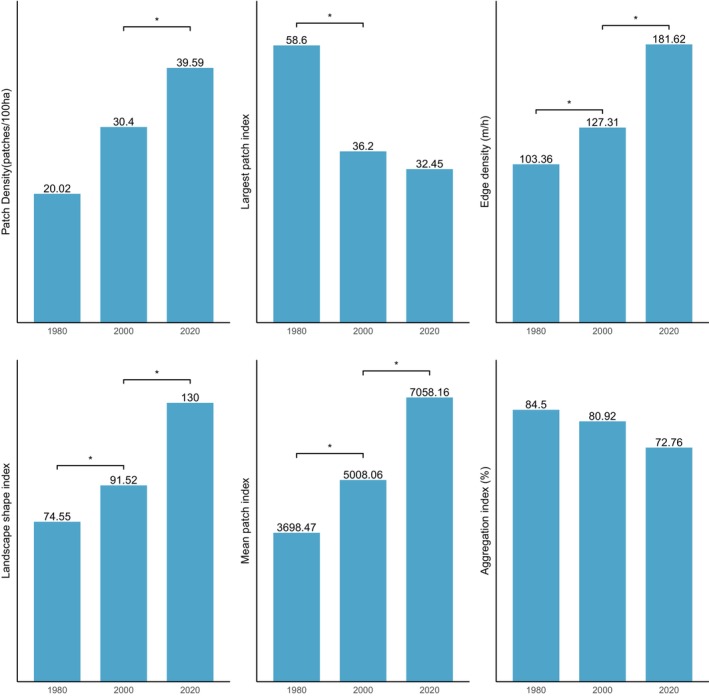
Changes in landscape structural metrics in the Bia conservation areas (BCA) from 1980 to 2020. The bars indicate the results of the Wilcoxon signed‐rank test. * show statistically significant differences in landscape metrics at *p* < 0.05.

**FIGURE 9:**
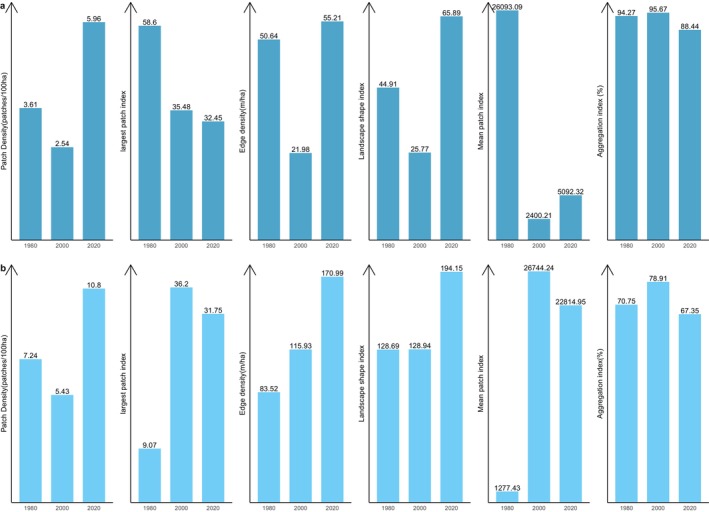
Structural changes in the closed‐canopy and open‐canopy forest classes in the BCA from 1980 to 2020. The set (a) closed‐forest class and (b) open‐forest class.

**FIGURE 10:**
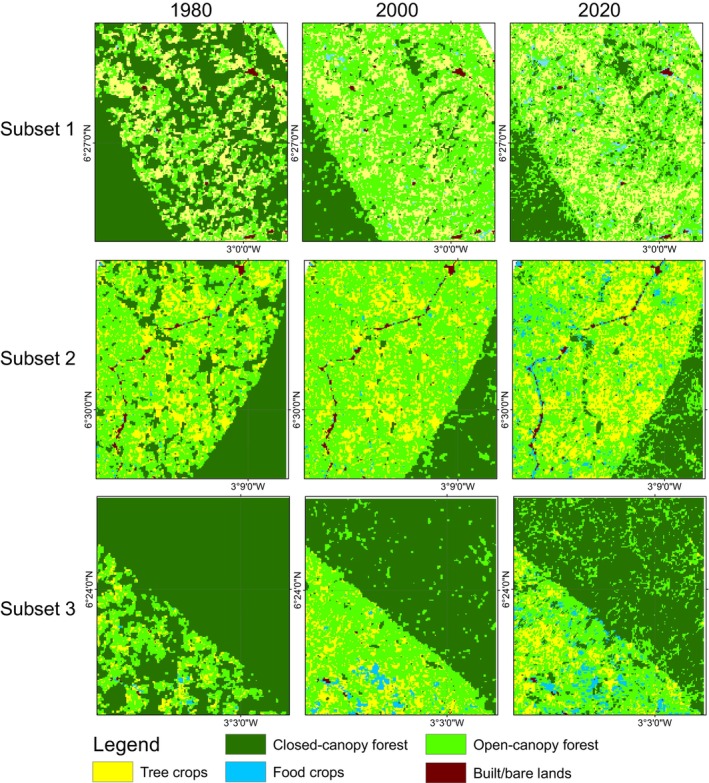
Reduction and fragmentation of forest in the ACA between 1980 and 2020 mainly driven by anthropogenic agricultural expansion.

Likewise, the BCA also experience fragmentation, with the focal patches becoming isolated, complex and irregular in shape (Figure 8: Figure 10). The PD, ED, LSI and MPI increased significantly between 1980 and 2020. The increase in PD from 28.02 patches/100 ha to 39.59 patches/100 ha shows that the landscape is subdivided into smaller fragments, increasing the number of patches per unit area. This is corroborated by the decrease in AI from 84.9% to 72.76%, implying increasing fragmentation. At the class level, the forest classes, that is, the closed‐canopy and open‐canopy forest classes, also experience some levels of fragmentation. The closed‐forest patch continued to fragment into isolated patches, as indicated by the increased PD and declined AI (Figures 9 and 10). The decline in the LPI from 58.6 to 32.45 indicates that the largest closed‐forest patch is getting smaller, indicating a loss of contiguous closed‐forest habitat (Figure 8). The significant drop in the MPI for the closed‐forest patch means that, on average, the close‐canopy forest classes are further apart, indicating increased patch isolation. A visual inspection of the changes (Figure 10) in the landscape shows the fragmentation and disruption of forest habitat, loss of contiguous habitats and reduced patch size. The results (Figures 9 and 10) also point to a complex and irregular landscape and reduced connectivity amongst forest habitat classes.

## 4 Discussion

### 4.1 Changes in Total Forest Area and Landscape Fragmentation in the ACA and BCA

The research revealed a significant loss in forest habitat to agricultural expansion in the off‐reserve regions of the study area within the 40‐year study period. This finding is confirmed in a related study by Abu et al. (2021), who observed that the concentration of cocoa farms is higher and more concentrated within the 5 km buffer of the protected areas in Ghana. The observed conversion of forests to cocoa and other crops in the off‐reserve areas could be attributed to the significance of ‘forest rent’ whereby farmers utilise forest soils as a source of nutrients to enhance agricultural productivity (Ashiagbor et al. 2022). Also, as human populations grow, human activities continue to increase in the landscapes, increasing LULC conversions (Belete, Maryo, and Teka 2023; Rawat et al. 2021). Gockowski and Sonwa (2008) and Ajagun et al. (2021) observed that there are no longer large tracks of uncultivated land readily available for development by cocoa farmers and other tree crops, except for forest lands. Consequently, the expansion of these activities is bound to be at the expense of Ghana's tropical forest (Ajagun et al. 2021; Gockowski and Sonwa 2008).

The study found that despite increased forest conversion to agriculture within the 5 km buffer zone of the conservation areas, the ACA and BCA have benefitted from strong protection measures, as no agricultural encroachments were observed within their boundaries. This suggests that the management strategies of these protected areas have been effective (Bailey et al. 2016). This finding corroborates the study by Naughton‐Treves, Holland, and Brandon (2005), confirming that parks are generally effective at curbing deforestation within their boundaries. Furthermore, Abu et al. (2021) investigated encroachment in conservation areas in Ghana and found no evidence of cocoa encroachment in the ACA and BCA. This may be attributed to the strict protective measures the Ghana Forestry Commission implements, which prevent encroachment.

However, the absence of agricultural encroachment within the boundaries of these conservation areas does not necessarily mean alleviating biodiversity threats or guaranteeing resident fauna (Bailey et al. 2016; Redford 1992). It has been observed that trees often remain in forests where the fauna has been depleted by poaching and hunting, as highlighted by Redford (1992). Holbech et al. (2018) reported that between 1990 and 2009, there was a decline in hornbill abundance in Ankasa by 39% and in Bia by 72%. They specifically reported that the IUCN Vulnerable Upper Guinea endemic Yellow‐casqued Hornbill ( *Ceratogymna elata* ) was only detected in Ankasa, even though this species was once common in Bia and the Western Region, especially in Ankasa in the early 1990s. Similarly, the abundance of the Black‐casqued Hornbill ( *Ceratogymna atrata* ) and the Western Piping Hornbill ( *Bycanistes fistulator* ) also declined by 61% and 85%, respectively, by 2009 compared to 1995. In addition, Oates et al. (2000) reported the extinction of Miss Waldron's red colobus monkey (*Procolobus badius waldroni*). This primate species was historically relatively abundant in the Bia and Ankasa landscapes.

Generally, the results of the fragmentation analysis point out that the forest patches outside the boundaries of BCA and ACA have become fragmented, disjointed and significantly reduced in size. This observed change was driven primarily by agricultural expansion, mainly cocoa, in both study areas. Agricultural commodities such as cocoa, oil palm and rubber are the most prevalent drivers of landscape transformation in Ghana and Cote d'Ivoire (Afriyie et al. 2021). Agriculture has been observed and discussed in the literature as the primary driver of landscape changes in Ghana (Kleemann et al. 2017; Manzoor et al. 2022). In both Ankasa and Bia, cocoa dominated and drove the transformation in the structure of the landscape in the once forest‐dominated landscape.

### 4.2 Implications of Forest Cover Loss and Fragmentation for Conservation of the Bia and Ankasa

These forest‐agricultural conversions in the buffer of the conservation areas can reduce the effective size of the conservation areas, reducing their ability to safeguard biodiversity and ecosystem functions (Bailey et al. 2016). Available scientific literature has related anthropogenic land use conversions around protected and conservation areas to human–wildlife conflict, bushmeat hunting, poaching and species extinction (Bailey et al. 2016; Meng et al. 2023). A total of 49 elephant crop damage occurrences involving 44 farms owned by 36 farmers were documented in a research by Sam et al. (2005) in the BCA. Various studies, including those by Sam et al. (2005) and Harich et al. (2013), have documented multiple conflicts between humans and wildlife in the landscape, and according to Sam et al. (2005), the frequency of raids increases as proximity of the farm to the park boundary reduces. According to Bempah, Dakwa, and Monney (2019), habitat loss through forest conversions to cocoa and other agricultural land uses has reduced the average species encounter rate from 10.2/km in 2008 to 3.7/km in 2016 in the ACA.

As indicated by the results, the conversion of open forests to cocoa and rubber could significantly impact the landscape's biodiversity. Cocoa and rubber, as monoculture crops, are reported in the literature to have low levels of biodiversity and significantly contribute to the loss of many species (Mang and Brodie 2015; Phommexay et al. 2011). The conversion of forest ecosystems to monoculture cocoa and rubber results in the destruction of diverse habitats and the displacement of various plant and animal species (Donald 2016). For example, the BCA has experienced significant biodiversity loss due to the expansion of cocoa. Daniel et al. (2018) found that converting forested areas into cocoa farms led to a decline in the abundance, richness and diversity of plant species within the BCA. Similarly, a report by the IUCN titled Protecting Ghana's Natural Heritage: A Biodiversity Assessment of Cocoa Farms within the Bia Conservation Area identified cocoa farming as a significant driver of biodiversity loss in the Bia region. This report highlighted the loss of critical forest habitats, the decline in plant and animal species and the disruption of ecological processes caused by cocoa expansion. These studies provide evidence that the expansion of cocoa, rubber and other monoculture plantations drives biodiversity loss and the overall degradation of ecosystems.

The immediate loss of forest cover in the off‐reserve landscape and the significant levels of forest fragmentation, resulting in the loss of forest connectivity, have significant implications for wildlife habitat loss and overall degradation (Laurance, Vasconcelos, and Lovejoy 2000; Cagnolo, Cabido, and Valladares 2006; Cote et al. 2017). A plethora of studies have demonstrated that the conversion of both closed and open canopy forests to other land uses has profound effects on crucial ecological processes such as changes in species composition, distribution, abundance, primary production and biotic interactions (Ebagnerin and François 2015; Muhammed and Elias 2021; USEPA 1999).

The patches of open‐canopy forest in the landscape are essential features, primarily ones found around conservation areas, as they serve as a habitat for some species, predominantly arboreal species in the area (Faria et al. 2006; Smith‐Ramirez et al. 2010). These patches connect isolated areas of closed‐canopy forests, enhancing the habitat quality (USDA 2004). They also serve as corridors, linking conservation areas to forest reserves and facilitating the movement of animals across the landscape, and also giving them access to a variety of habitat resources, which are often distributed across the landscape, enabling them to search for food and other essential resources (Collinge 1996; USDA 2004; McIntyre and Wiens 1999). However, the mobility of species is restricted by increased fragmentation in open‐canopy forests (Yates and Muzika 2006). As it becomes difficult for individuals to travel across habitat patches, there may be an increase in inbreeding and a loss of genetic diversity (Laurance, Vasconcelos, and Lovejoy 2000). As a result, the population becomes less healthy over time, which increases its susceptibility to disease and the likelihood of extinction (Benedick et al. 2006). For example, the loss of forest connectivity, mainly driven by cocoa expansion and logging, has been attributed to the population decline of the Roloway Guenon (*Cercopithecus diana roloway*) and the white‐naped mangabey ( *Cercocebus atys lunulatus* ), both of which are considered to be primarily restricted to the BCA (Asare et al. 2014; Oates, Struhsaker, and Whitesides 1997; Oates et al. 2000).

In 2000, the Wildlife Division of Ghana introduced the Community Resource Management Areas (CREMAs) programme, a Collaborative Community‐Based Wildlife Management policy, to address the many challenges associated with wildlife conservation and habitat conversions around the protected areas in Ghana. One of the goals of the programme was the community's willingness to reserve remnant forests outside protected areas and to regulate further agricultural expansion (Osei‐Owusu and Frimpong 2019). Despite this, these CREMAs have been characterised by a lack of participation and have yet to meet the implementation goals (Bempah, Dakwa, and Monney 2019).

Replacing monoculture cocoa with cocoa agroforestry in off‐reserve areas can enhance the connection between PAs and forest reserves, leading to more available habitats for wildlife (Asare et al. 2014). Critchley et al. (2022) pointed out that increasing tree coverage in cocoa‐growing regions in Ghana can help address fragmentation issues and improve forest connectivity. Cocoa agroforestry systems can potentially contribute to producing various ecosystem services compared to monoculture systems. Critchley et al. (2022) recommended prioritising the promotion of agroforestry systems that are more heavily shaded in regions near protected areas and conserved forests to facilitate the connection of habitats and the provision of ecosystem services.

The study did not consider the impacts of climate change, seasonal fluctuations, or other environmental factors that drive changes in the landscape. It also did not analyse the complex issues related to human–wildlife conflict, which are linked to human agricultural activities near conservation areas. Additionally, the study did not assess the effectiveness of conservation efforts or consider the perspectives and knowledge of local communities and conservationists. By recognising these limitations, managers of the ACA and BCA, as well as conservationists, can gain a better understanding of the complexities and challenges associated with conservation efforts in these areas.

## 5 Conclusion

This research gives an overview of changes in land use and landscape structural changes in the ACA and BCA and their catchment, revealing the degree of changes over 20 years. According to the study, the off‐reserve areas recorded most land use land cover changes, with cocoa dominating the forest conversions. It was also observed that the forest cover outside the PAs was fragmenting, mainly due to increasing agricultural activities. As populations are expected to grow, these trends may continue and lead to increased human–wildlife conflict, declines in habitat productivity and the overall degradation of the protected areas. Based on these findings, there is a need for immediate ecological restoration and conservation efforts to reduce landscape changes' potential impact on the wildlife population (fauna and flora) and vegetation cover. Ecologists have recommended transitioning monoculture cocoa systems to cocoa agroforestry systems to improve forest cover and forest connectivity within adjoining cocoa farms in the landscapes of these PAs. Also, managers and stakeholders must intensify interventions and continual engagements with CREMA communities to help achieve the programme's overall objectives.

## Author Contributions

**George Ashiagbor:** conceptualization (lead), formal analysis (lead), investigation (lead), methodology (equal), resources (equal), supervision (lead), writing – review and editing (equal). **Sinka Khadijah Abubakar:** formal analysis (equal), investigation (equal), methodology (equal), software (equal), writing – original draft (lead). **Sandra Sawdiatu Inusah:** formal analysis (equal), investigation (equal), methodology (equal), software (equal), writing – original draft (equal). **Abena Owusu Adjapong:** formal analysis (equal), methodology (equal), writing – review and editing (equal). **Gideon Nyamekye Osei:** investigation (equal), methodology (equal), software (equal), writing – original draft (equal). **Prosper Basommi Laari:** formal analysis (equal), resources (equal), writing – review and editing (equal).

## Conflicts of Interest

The authors declare no conflicts of interest.

## Data Availability

The Landsat satellite images utilised in this research are free to download on the USGS portal (https://earthexplorer.usgs.gov/). The GPS points from the field that were used to classify the satellite images into LULC maps can be accessed at https://doi.org/10.7910/DVN/Y3WWMB. The R script used for the random forest classification is available at https://doi.org/10.7910/DVN/1NGIVD.
